# Taking Charge: Social Support Dynamics among Older Adults and Their Significant Others in COVID-19 Vaccination and Mitigation Efforts

**DOI:** 10.3390/ijerph20064869

**Published:** 2023-03-10

**Authors:** Mark Brennan-Ing, Yiyi Wu, Jasmine A. Manalel, Ruth Finkelstein

**Affiliations:** Brookdale Center for Healthy Aging at Hunter College, The City University of New York, New York, NY 10035, USA; mi708@hunter.cuny.edu (M.B.-I.);

**Keywords:** older adults, COVID-19, social support, health behavior, health messaging

## Abstract

Older people have been disproportionately affected by the COVID-19 pandemic and are often portrayed as passive victims of this global health crisis. However, older adults do take responsibility for their own health and that of others in large part through social network dynamics. The purpose of this study was to understand the processes whereby older adults’ social networks shape their own health behaviors, and vice versa, in the context of COVID-19 vaccination and other mitigation efforts. Qualitative data from 77 older adults between ages 65 and 94 obtained through focus groups or individual interview participants were analyzed. Participant narratives demonstrated the reciprocal nature of social support and health behaviors and provided evidence that COVID-19-related health behaviors in this population were motivated by social support, altruism, and life experience. These findings emphasize older adults’ active role as health promoters in their families and communities, keeping themselves and their significant others safe from COVID infection. Implications for the role of older adults in community health promotion efforts are discussed.

## 1. Introduction

Since the beginning of the COVID-19 pandemic, people have been talking about aging and older people in terms of risk of infection, disease severity, hospitalization, and risk of death. Some have pointed to age as a risk for severe COVID outcomes [1], with the initial outbreaks of COVID-19 infections in older adult congregate settings undoubtedly reinforcing this narrative [2]. More accurately, the risk of poor COVID outcomes is associated with underlying intrinsic and extrinsic age-associated factors that contribute to infection and disease progression. These factors include: immunosenescence, higher rates of comorbid conditions (i.e., hypertension, diabetes, and obesity), and living in congregate settings or multigenerational households [1,2,3].

Without question, the COVID-19 pandemic disproportionately impacted older adults in terms of greater disease severity and mortality [1,2,4]. Older people were also negatively affected by COVID mitigation measures, such as lockdowns and social distancing, which increased social isolation, loneliness, and contributed to interruptions in the receipt of vital health and social services during the first years of the pandemic [2,3,4]. However, despite all the talk about aging, largely lacking in this narrative are the voices of older adults themselves and understanding how this population responded to the myriad threats of COVID-19. In this paper, we examine how older adults played an active role in responding to the pandemic rather than being passive victims in the greatest health crisis in generations and how this was expressed through interactions with their social networks. 

### 1.1. The Intersection of Ageism and COVID-19

Ageism, namely negative stereotyping and discrimination related to chronological age, is fundamental to understanding how older people were affected by and responded to the COVID-19 pandemic. Our society largely focuses on the negative age stereotypes about growing older such as poor health creating a drain on healthcare resources, a lack of meaningful social contributions after retirement, and old age being a life stage of loneliness, dependency, and decline [5,6,7,8]. Ageism exacerbated the impact of the COVID-19 pandemic on the older population [9,10]. Because of the greater severity of COVID in older adults, resentments around COVID mitigation measures including social distancing, lockdowns, and quarantine were focused on older people. Older adults were perceived to be the reason for needing these strategies because they were “vulnerable” and “needed protection” [9,10,11,12,13]. Ageism related to COVID also amplified the negative effects of the pandemic on the physical and emotional well-being of older adults to the extent that they internalized ageist stereotypes [11,14,15]. This reinforced paternalistic attitudes towards an older population [11,12,13]. 

However, were these perceptions congruent with the actual lived experience of older adults during the pandemic? The unthinking adoption of such ageist and paternalistic attitudes ignores the reality that older people are those in our society with the most lived experience and are sources of generational knowledge and wisdom while continuing to make positive social contributions in numerous ways [11,12]. One must consider the extent to which older adults were not helpless victims of the COVID pandemic, but rather played an active role in protecting themselves and others in their social networks through infection mitigation and vaccine promotion. The conceptual framework of health locus of control is useful in understanding the internal motivations for assuming responsibility for one’s own health that guided these social network interactions. 

### 1.2. Health Locus of Control and COVID-19

Health locus of control (HLoC) refers to how individuals perceive that their life and health are under their own control (internal LoC) versus controlled by external forces (external HLoC) [16]. People with high internal LoC believe that health outcomes are primarily contingent on their own choices and behaviors. Individuals with high external LoC believe that health is beyond their own control, determined by “powerful others” such as healthcare professionals, unpredictable events, and luck or fate [17]. Importantly, HLoC falls on a spectrum; people have varying degrees of both internal and external HLoC and health behaviors are best understood as interactions between these two dimensions of health beliefs [18].

In theory, external HLoC might prompt older adults to listen to healthcare professionals and other authority figures and to comply with government-mandated COVID mitigation measures, ultimately resulting in better health outcomes. However, external HLoC is positively associated with symptoms of depression and anxiety, while internal HLoC is found to be associated with adherence to COVID mitigation measures [19,20,21]. The COVID-19 pandemic was less stressful for those with greater internal LoC who believed that they have influence over their own lives, even during a time of crisis, compared to those with greater external LoC [22,23]. 

### 1.3. Older Adults, Social Networks, and Health Behaviors

Research also finds associations between LoC and social support resources. Individuals with greater internal LoC also tend to have more sources of social support that promote positive health behaviors [24]. 

Older people do not consume health information or make decisions about COVID-related health behaviors in a vacuum, but rather in the context of close social ties. Social relationships affect health across the lifespan [25,26,27] reflecting reciprocal influences on health behaviors. Close friends and family can encourage and motivate health-promoting behaviors, such as exercise and engagement in care, as well as harmful ones, such as drinking alcohol and poor dietary habits. Likewise, older adults influence the adoption of health behaviors of their close peers; however, this tendency towards behavioral homophily (i.e., sharing behaviors with those similar to you, for example, in age) may be more pronounced for poor health behaviors, such as physical inactivity [28]. 

Families are an important source for developing, adopting, and changing health beliefs and behaviors as well. Older adults are just as often the promoters of positive health behaviors as they are recipients of health encouragement from same-age and younger relatives. There is an extensive body of work documenting the influence that older spouses have on each other’s health and health behaviors, such as chronic disease management and care engagement, e.g., [29]. Intergenerational support exchanges, such as encouragement of healthy eating behaviors [30], are another important context for the bidirectional influences that older adults have on their family members, particularly during the COVID-19 pandemic [31]. Grandparents, in particular, have long been a source of health information and behaviors for families, especially those in multigenerational households where they play a central role in caring for grandchildren [32]. 

### 1.4. Purpose and Rationale

The ability of older adults to successfully weather the COVID-19 pandemic was associated in part with their ability to activate social network resources. However, our understanding of health behaviors and social network dynamics in older populations suggests that older adults take responsibility for their own health as well as shaping the health behaviors of others by acting as sources of information, advice, and generational wisdom. In the current study, we sought to understand these processes in the context of COVID-19 vaccination and mitigation efforts and efforts to cope with the impact of the pandemic.

## 2. Methods

Data were obtained from a larger qualitative study of how race and ethnicity were associated with older adult attitudes toward COVID-19 vaccination, mitigation efforts, and sources of trusted health information in partnership with one of the largest senior service providers in New York City. Since we were interested in better understanding how people were thinking about a novel and emerging phenomenon—the COVID pandemic and its influences on their decision-making—a qualitative approach was the most appropriate method to understand these complex phenomena.

### 2.1. Source of Data and Procedures

While our preferred data collection method was using focus groups in order to obtain additional information gained by group interaction, we designed the study with the flexibility to employ individual interviews as necessary due to COVID restrictions or recruitment complexities. In line with the aims of the larger study, we recruited from six prominent linguistic/demographic groups of older adults in New York City, namely, U.S.-born Black/African Americans, West Indian-born Blacks, Spanish-speakers, Chinese-speakers, U.S.-born Whites, and Russian-speakers. Recruitment flyers in the languages most commonly spoken at specific program sites (English, Spanish, Mandarin Chinese, and Russian) were posted by staff at the senior service sites (older adult residences and, senior centers). We also worked with the organization’s vaccine coordinator to organize and conduct thirteen tabling events at service sites to screen and schedule potential participants. Recruitment and data collection occurred from October 2021 to January 2022. 

Older adults interested in participating were screened for eligibility: (1) age 65 or older; (2) able to participate in a 90-min in-person focus group discussion or telephone interview; (3) willing to discuss COVID-19 vaccination; and (4) able to converse in English, Spanish, Russian, or Mandarin Chinese. Eligible older adults were scheduled into focus groups or individual phone interviews. Interviews were conducted when a person was eligible but the focus group time/place was not convenient or COVID mitigation protocols precluded in-person gatherings. Participants provided informed consent. Research materials, including informed consent, were available in English, Russian, Spanish, and Mandarin Chinese. Non-English language focus groups and interviews were conducted by research staff who were either native speakers or fluent in those languages. Focus groups and telephone interviews were audio recorded, with permission. We developed a discussion and interview guide that covered general sources of health information, experiences with vaccination, COVID-19-vaccine-specific concerns and motivators, the vaccination process, and overall experiences during the COVID-19 pandemic. The study protocol was approved by the City University of New York Integrated Institutional Review Board. The participants received a USD 20 gift card as compensation for their time.

### 2.2. Participants

Seventy-seven older adults participated in focus groups (*n* = 62) or interviews (*n* = 15). The largest group was the U.S.-born Blacks (*n* = 22), followed by Hispanic/Latinx older adults (*n* = 21), Russian speakers (*n* = 11), Chinese speakers (*n* = 9), U.S.-born Whites (*n* = 8), and West Indians (*n* = 6) (Table 1). Most participants were women, though there was at least one man in each demographic group except for the West Indians. The sample age ranged from 65 to 94 with most in their 70s. The majority lived alone, though a few lived with children or spouses. Educational background varied, but many said they had some college attendance or were college graduates. Most were clients of the senior service provider, although a few were friends or aides of residents who met the eligibility criteria. Since the senior service provider only serves low-income older adults, almost all the participants were low-income. Most of the participants stated that they had received COVID vaccines at the time of the interviews.

### 2.3. Qualitative Analysis

Audio recordings were uploaded onto secured, password-protected network drives and encrypted. English-language audio recordings were transcribed using Descript software (Version 41.1.0, https://www.descript.com/, accessed on 24 January 2023). Audio recordings in Chinese, Russian, and Spanish were translated and transcribed either in-house or via a third-party vendor. The ranscriptions were analyzed using the Atlas.ti 22 qualitative software package (https://atlasti.com/; accessed on 24 January 2023). The research team developed and finalized a project codebook via an iterative process. The initial codebook was developed deductively from the interview guide. This codebook was then refined inductively, from the data, after an initial coding round of each of the focus groups. Memos were used extensively to document impressions of the data, the adequacy of the coding framework, and emergent themes. The research team met regularly throughout the coding process to discuss the preliminary findings, coding modifications, and interpretation of the narrative data. Each transcript was coded independently by two members of the research team using the final codebook. Disagreements were resolved by discussion.

The documents were analyzed for themes that emerged from the qualitative analysis coding (see Table 2). These codes were then mapped onto potential frameworks, illustrating thematic flows. We sought to capture both the longitudinal aspect of older adults’ lived experience during the pandemic with a focus on decisions concerning vaccination in the context of interpersonal and social factors. In the current paper, we focus on themes that emerged regarding social support dynamics and health behaviors. 

### 2.4. Findings: Social Supports Encouraged Vaccinations

Consistent with our understanding of the nature of social support and health behaviors, our participants shared stories about how their social networks played a role in shaping their decisions around COVID vaccination and other mitigation measures. These narratives provide evidence that many older adults were operating in well-established social networks developed over their lifetimes. Both family and friend network members influenced our participant’s decisions to get vaccinated, including direct support and indirect guidance.

*And my daughter that lives in North Carolina called me to say, “Mom, I took it. I think you need to take it too”. So those was [sic] the factors that point [sic] me in the direction to go ahead and take the vaccine.*

*In the beginning, I wasn’t [convinced]. And then I had a friend, she convinced me, um, also to take it.*

Some participants were more hesitant to get vaccinated than others, especially those with low trust in the media, government, and medical authorities. However, such distrust did not always result in an immediate and firm refusal to take the shot. Instead, some participants reached out to their networks to engage in conversations, ask questions, interrogate ideas, and assess health risks and eventually make their own decisions about getting vaccinated.

*Well, the first time I heard about it was on TV. So I said to myself, I’m not putting that in my body. I’m not doing that. So one of my nurses, my niece is a nurse, registered nurse, and she called me and she told me, you don’t have to worry about it… So I said, I’ll think about it. So what I did, I called my two sons and we did like a three-way on the phone. I don’t know how to do it, but they did it… everybody’s dying from it and everybody’s getting sick and all my nieces and nephews, because my mother had 13 children. I’m number 12. So we all got together on the phones and stuff like that. And on this, you have to get it, you know, we don’t want anything to happen to you. Yeah. And my two sons, we all got together and I said, okay, I’m going to do it. So we had it done here at the center, the first and second shot.*

Some other strategies included critical observation of their peers’ experiences with the COVID vaccine. After engaging in conversations with her peers, one participant took her vaccines, explaining:

*Everybody around me was getting the vaccine. I was the only one, if they’re denying and everybody else went and got it and nothing happened to them. And I said, well, let me try then.*

### 2.5. Evaluation of Vaccine Information and Motivations for Taking the Jab

Concerns about how the vaccine was developed and potential side effects were expressed by some of our participants, which also contributed to the initial vaccine hesitation for some.

*Well, when I first heard of the vaccine, I had mixed feelings. I was worried about, like so many other people, about how fast it was developed.*

*There were many tales and myths about the COVID vaccines at that time. A lot of different experts at the time would say different things. At first, an expert said something could go wrong with the vaccine. The second expert said that unless the body is ill, it is fine. There is no certainty.*

*They said after the third dose, your genes might be altered. We know so little about it. Even if it is actually mutating our genes, there is nothing we could do since we already got our shots, ha ha ha. We know so little about genes. What could we do?*

However, such concerns and fears did not stop many from actively coming up with creative strategies to mitigate potential vaccination risks. A couple we interviewed who were both in their late seventies created a lagged vaccination plan to minimize potential risks and maximize safety. The wife related:

*I was worried. I told him that the two of us should get the dose separately. So if you’re not feeling well, I can take care of you. If I take the shot first and I’m not feeling well, you can take care of me. He said he would take it first. Then you should do it later. All I am saying is that if two people took the shot together and they both felt sick, they couldn’t take care of each other. They would lay down and suffer.*

For many of our older participants, the decision to get vaccinated was rooted in a spirit of altruism. While there may have been significant concerns about vaccine safety and side effects, many believed that it was their obligation to get vaccinated to protect their community and the younger generation. One participant was initially reluctant to get vaccinated because of her underlying health conditions. As she recounted, even her doctor could not give her a clear answer as to whether it was a good decision to get vaccinated at the time. She explained that she decided to get vaccinated because:

*To protect the younger people. We old people are fine with dying. I have lived long enough. I just do not want to infect other people. That was why I chose to get vaccinated.*

During a time when misinformation and disinformation about vaccine safety were being widely circulated in the media, some participants sought guidance from their past life experiences, which eventually informed their decision to get vaccinated and protect those around them. For example, one told us she “had mixed feelings” when she first heard about the COVID vaccine. Like many others, she was skeptical of the speed of its development. However, she gradually relieved her concerns as she recalled her past experiences working in the healthcare industry:

*I’ve taken vaccine for hepatitis because I was working in healthcare and hepatitis many years ago was a high risk for people working in, in a health profession. And we were vaccinated to guard against contracting, um, hepatitis. And also as an adult, I’ve taken vaccines for pneumococcal infection, so to defend that, so I’ve taken the flu vaccine as well. So I said to myself right now, there’s no cure for what’s going on right now. And the only way that I could see me safeguarding myself or protecting myself and my mother was supposed to be vaccinated. And that’s why I convinced myself that it’s worth the risk.*

In fact, in our study, the desire to “protect others” was identified as one of the top motivators for many to take the jab. Another participant told us:

*We older people are fine with dying. However, I do not want to go to public places to infect others. I do not want to infect the living people before I die.*

Sometimes the decision to get vaccinated was expressed with a degree of indignation, driven by their strong desire to continue serving their community:

*I didn’t think twice about it because I know that in my volunteer work, I go places when I deal with the homeless population and I wanted to continue my volunteer work. So I know that I was going to, there was some hesitancy. I was going to take that vaccine because viruses, they don’t stay the same.*

Moreover, protecting others is also the motivation for many to persistently comply with other COVID mitigation protocols, such as the mask mandate and social distancing:

*Why do I wear a mask? Not just to protect me, but to protect the people around me. Um, you know, too people and... too many people is making it about themselves and not about what it is, you know? And sometimes I have to remind people when I’m talking to them, this is not just a right here thing. It’s an all-over-the-world thing. Remember the whole world was shut down. You know, this is not just about you or us. It’s about them.*

### 2.6. Stepping Forward: Older Adults as Health Advocates and Sources of Support

Due to government prioritization, older adults (as well as the health and direct care workforce interacting with them daily) had access to COVID vaccines earlier than younger people, including their own family members. Many of our participants became vaccine advocates within their own networks based upon their experience as early adopters, their constant exposure to the stream of vaccine information and misinformation, their personal stake in needing others to get vaccinated to protect them, and their role as trusted advisers within their social networks. This role, which came through strongly in almost every group and interview, stands in sharp contrast to the dominant ageist image of older adults as confused victims of COVID. Meanwhile, these older people were exceptional in building trust in a pandemic of distrust, enabling effective and selective information dissemination and helping shape social norms within their networks and communities. 

However, some participants shared that their children and other younger family members were unwilling to get vaccinated due to concerns about vaccine safety and efficacy at the time they became eligible for the shot. In response to such hesitancy, our older participants shared vivid stories about how they took the role of health advisors for their families and advocated for the COVID vaccine.

*My youngest son wasn’t going to do it. And I remind him, “you have asthma, baby, you know. That virus hit your lungs, you don’t know what happened to you. Do you know, your asthma, baby, you used to smoke, you don’t know the condition of your lungs. Now don’t do that to yourself. You know?”*

Some participants use their own experiences with vaccination and COVID as examples to facilitate their families’ decisions:

*My son has not taken the vaccine up until now… Everybody’s took it [sic], but he refused to take it. [He said] “Oh mommy, you took it and you got COVID twice” and I say, “Okay, well maybe if you take it, you will get it three times. I got it twice, if you take it, you will get it three times. But as I said it worked in a great way and because I’m still alive today. I got it twice and I survived it. And here I am, and it was because of the vaccine”.*

Another participant told us:

*We guided my son so that he could take the vaccine. He’s, he’s not that old, but he’s not that young either, 30-something, 37. Uh, and he was pretty reluctant, right? But when we got vaccinated and we told him that the vaccine is just like giving you get a vaccine like, like any other. He got vaccinated a few months ago.*

Some participants convinced their family members by making vaccination a vital personal matter. One woman shared this story:

*And when I asked him [her son] whether he was going to take the vaccine or not, his thing was to me, he said, “Mom, I don’t think so.” And I said, “Why not?” And he gave me some crazy excuse, and I said, “Wait a minute… listen, that does not make sense to me. Think about it… because God forbid that you should contract the virus. I would not be able even to come and see you. It wouldn’t be even worth me coming to where you live to call me because I couldn’t see you. They’re not going to let me come in upstairs. And the same thing with myself.” And I said, “I will not want anything to ever separate us from not being able to be together”.*

Sometimes these conversations spanned many generations:

*My grandson, he’s in college, down in Alabama. He made me so angry with him. And he did contract COVID while he was in college, you know? So I made him promise me that he would take the vaccine. So what does he do? He said, “Grandma, I’ll always keep my promises with you, but this was one I’m not going to do.” So I said this to him. “Then if you get sick don’t come nowhere near me, you know?” And he said, “Grandma”, I said, “No, no.” I said, “I really mean that, you know?” And so now he told me to go, “Grandma, I’ll think about it”.*

In addition, participants’ active engagement in health promotion was not limited to vaccine advocacy. Since many of them lived in senior apartments, some also stepped forward to reinforce the mask mandate in the shared common area to ensure the safety of themselves and many other older residents:

*I went the other day in the elevator and came to the other floor. A young fellow did not wear a mask. I ask[ed] if he was a worker from here. He said yes... I told him you have to use masks in the hallway, in the elevator. “Okay, for you.” I offered him masks. He opened the package and he took the mask.*

### 2.7. Ambassadors of Altruism

As mentioned before, many participants shared altruistic motivations to get vaccinated because they highly valued the safety and lives of the younger generation and other community members. Some of them also transmitted these values to younger members of the community:

*It was one person I asked her and she said, “I’m not going. I’m not going.” I said, “Well, if you was still working at the nursing home, you would have to take it or you lose your job! You’ve got to protect the residents there”.*

In addition, as described earlier, peers played an important role in the decision-making process for many by delivering effective, tailored, and relatable COVID-related information. Many of our participants who once obtained support from peers paid it forward by becoming peer support for many others.

*Do the best we can. Those who are our friends, we let them know that this is a safe way, that’s the way to go.*

*There were still some that were hesitant, it was quite a few then was waiting to see. They weren’t all jumping to get it and I said, I explained to them that I already took it and that I’m feeling fine and, and some of them were convinced to go ahead and, you know, take the vaccine.*

Some expressed their vaccine advocacy among members of their faith communities:

*I have been to the Middle East, India, Pakistan, the Holy Land. I always had to take shots. And I am telling all the people—I am telling my Muslim counterparts—when you go to the Holy Land, you need to take shots*

Other participants’ advocacy specifically targeted the spread of misinformation:

*I’m going to tell you if I come across anybody who’s talking nonsense about the vaccine “Shut your mouth go take your vaccine and protect yourself.” I’ve heard concerns about people talking about turning monkey and donkey at this one and nonsense, you know, all over the place. Why? Take a vaccine, take the vaccine if it’s, if it’s beneficial to you. They wouldn’t call me to tell me nonsense like that because I won’t entertain it, you know? I wouldn’t, I would come meet with you at [the] door. Go to you, have to take a vaccine.*

Our participants shared that such messaging was often extraordinarily effective. This is because they were often well-respected and established members of their families and communities.

Moreover, the participants also described helping one another to find vaccination sites when vaccines were scarce and available appointments were hard to identify by providing information and logistical support. One participant described helping his friend with limited computer literacy to schedule an online appointment:

*Well, I have a friend who said that he really wanted to get vaccinated, but where they live is not as good as we are here. They asked me to help them register on the website and get vaccinated… They don’t know how to use the Internet. I taught them.*

## 3. Discussion

As with the rest of the population, older adults experienced disruptions to all aspects of their lives due to COVID-19, including social interactions, physical activity, and accessing healthcare. Consistent with other studies [33,34] participants reported that these disruptions caused a number of negative emotions, such as frustration, fear, and anxiety rooted in worry about themselves and their loved ones. Many participants described the amount of information they had received during the pandemic as overwhelming. Much information was seemingly contradictory and confusing, which eroded their trust in the authority’s ability to deliver accurate information and disturbed their confidence in health services. Consequently, many participants declared having had doubts about the vaccine’s safety and efficacy when the vaccine initially became available. However, contrary to the ageist narrative that older adults were helpless victims of the pandemic, our qualitative data revealed that these older adults were actively engaged in evaluating changing health information, had reclaimed autonomy over their health decisions in collaboration with their social networks, and served as valuable sources of information and advice for others, and especially for the younger generation. As one of the participants vividly put it, “…sometimes, we old people are more thoughtful and open-minded than some young people. Our thinking is ahead of theirs.” The older participants in our study once again prove themselves as great assets to society through civic engagement and mentorship. 

The bidirectional nature of social support among older adults and members of their social networks is evident in the participants’ reports that they actively engaged in conversations about the vaccine. Several studies have documented changes in family and friend contact and support exchanges that older adults experienced during the pandemic [35,36], noting fluctuations in frequency, mode, and quality of exchanges. We observed that it was not only older adults’ own experiences that shaped their attitudes and behaviors regarding vaccination and other mitigation efforts, but also the experiences of their loved ones (e.g., family members contracting COVID-19). The participants in the present study noted the conversations they had with their adult children and grandchildren as being particularly important to making informed decisions about the COVID vaccine. In many instances, the pandemic brought families closer together. A recent study found that greater family cohesion, as well as overall social support, was significantly associated with COVID-19 prevention behaviors (e.g., social distancing and masking) [37]. What is largely underemphasized in this literature is the influence that older adults have on their families in terms of encouragement and support of health behaviors. 

Importantly, we observed that older adults served as key sources of information and health behavior encouragement for their families, peers, and communities during the pandemic. Specifically, they shared information about vaccination procedures in their communities, encouraged younger family members to get vaccinated, and endorsed other COVID-19 prevention behaviors, such as social distancing. There is a long tradition of utilizing older adults as peer educators in health promotion programs [38], including for nutrition, physical activity, and chronic disease management. There are several advantages to this model, particularly in the context of the pandemic or other health emergencies. For instance, older adults who have established community ties are more likely to have built rapport with the target groups, such that their messages for health promotion may be received more positively. In some social contexts, older adults may be viewed as having particular knowledge from their additional individual experiences, as well as sociocultural and historical events that they survived, e.g., wars or global economic crises [39]. While ageist stereotypes disrupt the ability to value the input of older people, when they speak from the perspective that they have a successful strategy to handle this new situation because they have encountered it before, this may be recognized as valuable. 

The leadership of older adults too often goes unrecognized. However, our findings exemplified the role that older adults take in influencing others in their social networks. However, the strategies employed to persuade older adults to adopt risk mitigation and vaccination relied heavily on public authorities (city and state health commissioners, Dr. Fauci, mayors, and governors) or recognized community leaders (ministers and leaders of community organizations). Neglected was amplifying the voices of people just like those the messages were trying to reach. Older adults could be effective messengers for public health campaigns and consult in the development of such campaigns. For example, it may have been helpful for senior serving organizations to include COVID-prevention messages recorded by their own members to play at the beginning of virtual classes. Similarly, ads depicting an older woman speaking directly to her peers about her decision-making process about taking the booster could be particularly salient for older adults, especially when considering the powerful tendencies towards behavioral homophily in peer networks [28]. 

Peers are widely recognized as effective public health messengers. Despite programs attempting to capitalize on older women’s role as keepers of wisdom (Promentoras, community health workers, and various community grandmother programs), interestingly, it is quite unusual for public health programs to identify older people as leaders among their peers. The present findings suggest they are effective in that role and that public health programs should include them as communicators in person, virtually, and in scripted campaigns. Despite some recognition of the value of leveraging older adults’ unique perspectives, experiences, and roles in society [39], there are still many public health applications that would benefit from the involvement of older adults. 

This study is not without limitations that could be addressed in future research. The study took place in New York City, where the general political and sociocultural norms towards vaccination are more positive than in many other parts of the U.S. and limit the generalizability of these findings. Although anyone over 65 and affiliated with the service organization at any level was eligible to participate in the research, due to the implication of the research topic in the recruitment flyers, recruitment might have been biased toward individuals who had more positive attitudes towards the COVID vaccine. For example, those who had not been vaccinated and/or strongly opposed vaccination might feel less inclined to participate, knowing that their opinions were less likely to be approved by others. Future research should examine the effects of social participation and network engagement on health decisions among older adults who had not been vaccinated or living in areas with low vaccination rates. 

Despite these limitations, this study provides unique insight into older adults’ roles in their families, peer groups, and communities during the pandemic. A key strength of the research is the diversity of the research sample, which encompasses a broad range of experiences by race, ethnicity, immigration experience, and age. Importantly, this study uniquely focused on older adults’ social support dynamics, proactive crisis management, and their critical role in health communication and advocacy during the COVID pandemic. More gerontological research is needed that centers on older adults’ social engagement and explores more ways for older adults to provide leadership in community public health initiatives. 

## 4. Conclusions

The findings in this study present a counter-narrative to the ageist stereotypes of older adults as helpless, vulnerable burdens to society during the COVID-19 pandemic. Older adults reported taking charge of their own health and that of their loved ones, motivated by both internal LoC, life experiences, and self-determination and external LoC, operating in social contexts. Importantly, the narratives presented in this research serve as compelling evidence against the prevailing political discourse that treats the growing aging population as a catastrophe rather than an opportunity. 

## Figures and Tables

**Table 1:** Participants’ characteristics by race and ethnicity.

Demographic Group	N	%
U.S.-Born Black	22	28
U.S.-Born White	8	10
West Indian/Caribbean	6	8
Chinese Speakers	9	12
Hispanic/Latinx	21	27
Russian Speakers	11	14
Total	77	100

**Table 2:** Findings’ themes and sub-themes.

Themes	Sub-Themes
Social Supports Encouraged Vaccinations	Social networks influence participants’ health decisions Engaging in conversations, asking for a second opinion, and making the right decision
Vaccination and Concern for Others’ Safety	Devising creative strategies to mitigate the potential vaccine side effects Using past life experiences to inform vaccine decision Protecting others
Stepping Forward: Older Adults as Health Advocates and Sources of Support	Using their own vaccine experience to inform families about vaccine safety Reinforcing other COVID mitigation protocols
Ambassadors of Altruism	Prioritizing the safety of others Engaging in reciprocal peer support Helping one another to locate vaccine resources

## Data Availability

Data are available upon reasonable request to the authors.
